# Diffuse hepatic and splenic Tc-99m MDP tracer uptake in case of multiple myeloma

**DOI:** 10.4103/0972-3919.72692

**Published:** 2010

**Authors:** Ashwani Sood, Rajeev Kumar Seam, Sandeep Sethi, Manish Gupta

**Affiliations:** Department of Radiotherapy and Oncology, Nuclear Medicine Center, Indira Gandhi Medical College, Shimla - 171 001, India

**Keywords:** Extraosseous uptake, multiple myeloma, Tc-99m methylene diphosphonate bone scan

## Abstract

Tc-99m methylene diphosphonate (MDP) bone scintigraphy has long been used for the evaluation of benign as well as malignant skeletal conditions. However, non-osseous tracer uptake on a bone scan is an unusual finding. There is a need to understand the pathophysiological basis of the non-osseous uptake, which may have a clinical relevance or deteriorating effect on the quality of the bone scan. We describe a case of multiple myeloma, where extraosseous uptake in the form of diffuse hepatic and splenic uptake, with almost normal skeletal tracer distribution, has been seen on the bone scan.

## INTRODUCTION

Bone scintigraphy is an important modality for the examination of various pathological conditions of the skeletal system. The extraosseous uptake is often an unexpected finding on the bone scan. There are several causes for such an uptake, although the responsible pathological entity is not always clear. The authors present a case of multiple myeloma, where a bone scintigraphy demonstrated intense hepatic and splenic uptake of the Tc-99m Methylene Diphosphonate (MDP) tracer uptake. This article reviews several possible reasons for such an uptake, and the exact cause in the present case may be one of them.

## CASE REPORT

**Figure 1 a and b:**
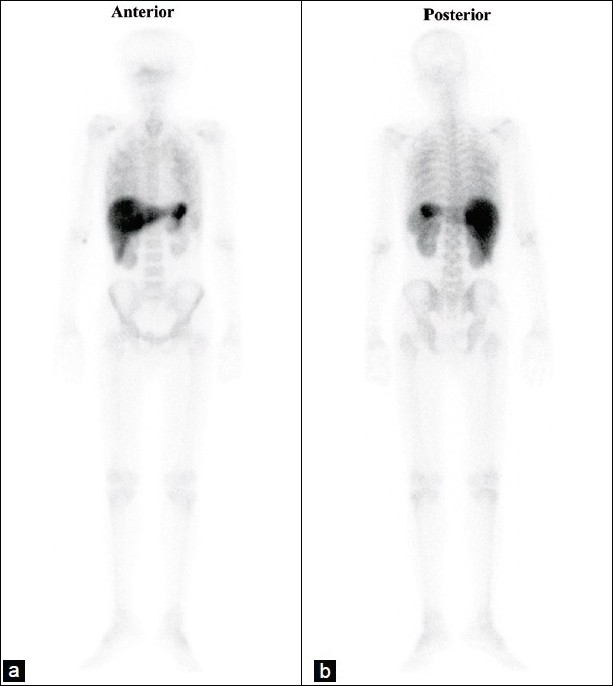
Anterior and posterior whole body bone scan showing diffuse and intense tracer uptake in the liver and spleen, without any obvious abnormality in the skeletal system

A 48-year old male presented with generalized body aches, a recent onset of severe pain in the upper portion of the back, and unexplained weakness. He was treated for pain and weakness with anti-inflammatory drugs and oral iron therapy outside, before presenting to our institution. The x-ray thoracic vertebral region revealed thoracic vertebral collapse at the D-4 level. Magnetic resonance imaging of that area also showed a D-4 vertebral collapse, with no other abnormal findings. The patient underwent a whole body bone scan for the evaluation of possible metastatic bone disease, with a differential diagnosis of multiple myeloma; 20 mCi (740 MBq) of Tc-99m MDP was administered and imaging was done in the anterior and posterior projections after an interval of four hours. The bone scan revealed diffuse and intense tracer uptake in the liver, spleen, and bilateral kidneys (Figure 1a and b). There was no tracer seen in the urinary bladder. The thoracic vertebral collapse seen on the x-ray and MRI was not visualized on the bone scan, either due to intense tracer activity in its vicinity or there was no osteoblastic activity in the diseased vertebra.

Other investigations included serum electrophoresis showing M-band (48.4%) and 60% plasma cells on bone marrow aspiration cytology, establishing the diagnosis of multiple myeloma. The serum electrolyte, calcium, phosphate, and parathyroid hormone levels were within normal limits. His renal functions were deranged initially, although they improved marginally with appropriate treatment.

## DISCUSSION

Bone scintigraphy is usually performed for metastatic bone disease, and it is generally not an indication in multiple myeloma, as osteolytic lesions characteristically show no tracer uptake.[1] Yet, a significant number of multiple myeloma patients undergo bone scintigraphy due to their presentation mimicking a metastatic bone disease. Non-osseous uptake of bone-seeking radiopharmaceuticals is seen in various conditions involving almost any organ. There is a need to recognize the pathophysiological basis underlying such an uptake. Faulty radiopharmaceutical preparations may be the cause of abnormal distribution, due to the formation of a Tc-99m colloid complex.[2] Palmer *et al*., reported that the colloidal preparation of Tc-99m hydroxymethylene diphosphonate may aggregate *in vitro* in the presence of calcium salts, which may be cleared by a reticuloendothelial system in the body.[3] In our case, bone scans performed on other patients on that day did not show any non-osseous uptake, thereby ruling out the possibility of faulty preparation.

Hepatic uptake has been observed in patients undergoing bone scinitigraphy, and focal uptake is more frequent than diffuse hepatic activity. Focal tracer uptake is usually seen in the case of hepatic metastasis, while intense and diffuse liver uptake of the tracer has been reported in the case of severe hepatic necrosis. Diffuse and intense hepatic uptake, followed by hypoxia due to respiratory failure, along with the development of hepatic necrosis, has been reported on a Tc-99m-hydroxy-methane-diphosphonate (HMDP) bone scan.[4] Splenic accumulation of the tracer had been observed in patients with alcoholic cirrhosis of the liver.[5] The patients who have undergone orthotopic liver transplantation have demonstrated hepatic and other ectopic soft tissue calcifications. Munoz *et al*., in their study of 20 patients undergoing liver transplantation, attributed hyperparathyroidism, calcium administration during and after surgery, renal failure, acid-base change, and citrate in fresh frozen plasma, as some of the potential pathological factors for such an uptake.[6] Our patient’s clinical presentation, ultrasound abdomen, and other investigations were not consistent with hepatic metastasis, necrosis, alcoholic liver disease or history of liver transplantation.

Several cases of diffuse hepatic uptake and decreased skeletal uptake on bone scan following an intravenous injection of iron colloid solutions for treatment have been reported. It is hypothesized that a Tc-99m iron-colloid complex is formed through transchealation of the MDP, yielding a compound with different organ affinity. This new agent is taken up in the Kupffer cells of the liver.[7] This does not appear to be the cause in our patient, who was treated briefly with oral iron therapy for diagnosed anemia.

Extra-skeletal uptake on bone scan, in cases of renal failure, has been demonstrated due to failure of excretion of the radiopharmaceuticals through the kidneys. Hypercalcemia secondary to chronic renal failure and hyperparathyroidism causes soft tissue microcalcification leading to extraosseous tracer uptake.[8] In hyperparathyroidism, the solubility product for calcium and phosphate may exceed greater than 60, causing a precipitation of calcium salts in the extracellular space, which may be reflected by the extraosseous uptake of MDP.[9] Similarly splenic accumulation of the bone agent has been seen in the scan of patients with sickle-cell disease. This uptake is assumed to be the result of splenic infarction and subsequent calcification.[10] Our patient’s clinical presentation and pertinent investigations ruled out these conditions for unusual tracer uptake in the liver and spleen.

Amyloidosis is often associated with deranged renal function in cases of multiple myeloma, due to light chain deposition in different organs.[8] Bone scans have been infrequently used to demonstrate solid organs and soft tissue involvement by an amyloid protein in patients with multiple myeloma. Jannsen *et al*., has showed that bone scintigraphy plays an important role in the evaluation of amyloidosis, by demonstrating the tracer uptake in the thyroid, tongue, salivary glands, nervous system, intestine, liver, spleen or kidney. In their study, five out of eighteen patients with biopsy-proven amyloidosis had bone agent uptake in the liver.[11] In a case report by Kanoh, a patient of multiple myeloma had shown soft tissue localization of a bone-seeking agent, which that was attributable to the presence of amyloid deposits.[12]

The probable presence of amyloidosis with deranged renal function, in this case, provides a possible explanation for the abnormal distribution of tracer in the liver and spleen, although histopathology was not done to establish the presence of amyloidosis. These findings may indicate a secondary effect of the disease, and recognition of such altered tracer distribution may provide important clinical information.
